# Circadian Gene Polymorphisms Associated with Breast Cancer Susceptibility

**DOI:** 10.3390/ijms20225704

**Published:** 2019-11-14

**Authors:** Monika Lesicka, Ewa Jabłońska, Edyta Wieczorek, Beata Pepłońska, Jolanta Gromadzińska, Barbara Seroczyńska, Leszek Kalinowski, Jarosław Skokowski, Edyta Reszka

**Affiliations:** 1 Department of Molecular Genetics and Epigenetics, Nofer Institute of Occupational Medicine, 91-348 Lodz, Poland; ewa.jablonska@imp.lodz.pl (E.J.); edyta.wieczorek@imp.lodz.pl (E.W.); edyta.reszka@imp.lodz.pl (E.R.); 2 Department of Environmental Epidemiology, Nofer Institute of Occupational Medicine, 91-348 Lodz, Poland; beata.peplonska@imp.lodz.pl; 3 Department of Biological and Environmental Monitoring, Nofer Institute of Occupational Medicine, 91-348 Lodz, Poland; jolanta.gromadzinska@imp.lodz.pl; 4 Department of Medical Laboratory Diagnostics and Bank of Frozen Tissues and Genetic Specimens, Medical University of Gdansk, 80-211 Gdansk, Poland; bastrzel@gumed.edu.pl (B.S.); lekal@gumed.edu.pl (L.K.); jskokowski@gumed.edu.pl (J.S.); 5 Biobanking and Biomolecular Resources Research Infrastructure (BBMRI.PL), 80-211 Gdansk, Poland; 6 Department of Surgical Oncology, Medical University of Gdansk, 80-214 Gdansk, Poland

**Keywords:** single nucleotide polymorphism, gene expression, circadian rhythm, circadian genes, breast cancer

## Abstract

Breast cancer (BC) is a major problem for civilization, manifested by continuously increasing morbidity and mortality among women worldwide. Core circadian genes may play an important role in cancer development and progression. To evaluate the effects of single nucleotide polymorphism (SNP) in circadian genes in BC risk, 16 functional SNPs were genotyped in 321 BC patients and 364 healthy women using the TaqMan fluorescence-labelled probes or High-Resolution Melt Curve technique in the Real-Time PCR system. The selected SNPs were analyzed for the risk of BC, progression, and the influence on gene expression in BC tissue pairs to demonstrate the functionality of genetic variants. The study showed a relationship between an increased BC risk under the dominant genetic model of *CRY2* rs10838524, *PER2* rs934945, and recessive genetic model of *PER1* rs2735611. A protective effect of *BMAL1* rs2279287 was observed among carriers with at least one variant allele. Moreover, we found an increased risk of estrogen-/progesterone-positive tumors under the dominant genetic model of *PER2* rs934945 and estrogen negative tumors under the variant genotype of *CRY2* rs10838524, *PER1* rs2735611. We demonstrated significantly altered gene expression of *BMAL1*, *CRY2*, *PER1*, *PER2*, *PER3* according to particular genotypes in the BC tissue pairs. Our findings support the hypothesized role of circadian genes in breast carcinogenesis and indicate probable biomarkers for breast cancer susceptibility.

## 1. Introduction

Despite significant progress in early diagnostics and modern therapies of BC in the last decades, breast cancer (BC) still constitutes the most common malignancy among women worldwide. According to the latest report of Global Cancer Statistics 2018 (GLOBOCAN 2018), breast cancer is ranked first place as a deadly disease among female cancer patients [1]. As the scientific environment is trying to find a new approach to elucidate the mechanism of mammary tumorigenesis, new biomarkers are needed to extend the diagnostics.

Single-nucleotide polymorphism (SNP) is the most common genetic variant in the human genome, which is considered a stable biomarker of genetic background to predict risk, progression, and treatment response to various diseases [2]. Researchers have revealed that several SNPs in circadian genes are associated with breast cancer susceptibility [3,4]. SNPs are located in different gene bodies including promoters, exons, introns as well as 5′- and 3′ UTRs [5]. Therefore, changes in gene expression and their cancer predisposition differ depending on the location of SNPs. The location of SNPs may affect gene expression by altering promoter activity, binding transcription factors, as well as DNA CpG sites methylation [2,6,7]. Additionally, cancer risk may depend on exonal SNPs by suppressing gene transcriptions and translations. SNPs in introns region are also not indifferent to gene functions. Such a location may generate splice variants of transcripts and promote or disrupt binding and function of long non-coding RNAs. SNPs in the 5′UTR affect translation, whereas SNPs in the 3′UTR affect binding of microRNA [2]. 

So far, there has not been enough research in relation to the incidence of breast cancer and the polymorphic variants of the circadian genes. Polymorphisms of positive *BMAL1*, *CLOCK*, *NPAS2*, and negative regulators of circadian rhythms *PERIOD* (*1*,*2*,*3*), *CRYPTOCHROME* (*1*,*2*), as well as *TIMELESS* have been most commonly studied. Only in individual epidemiological studies, statistically significant correlations between circadian gene variants and breast cancer have been demonstrated. Some of the analyzed SNPs have also been linked to other cancers and diseases [3]. Therefore, we conducted an association study among 321 newly diagnosed BC patients and 364 healthy women, living in the same longitude of Poland, in Gdansk and Lodz. The main objective of our project was to investigate an association between genetic variants of crucial circadian genes *CLOCK*, *NPAS2*, *BMAL1*, *PER1*, *2*, *3*, *CRY1*, *2*, and *TIMELESS* and the risk of BC, progression, and the influence of gene expression on BC tissue pairs to demonstrate their functional significance in the process of carcinogenesis in the mammary gland. 

## 2. Results 

The analysis was based on the cases of primary breast cancer patients and healthy volunteers, all of Caucasian ancestry. The selected characteristics of the study population are presented in Table 1. Among breast cancer patients, there were more non-smoking and pre-menopausal women compared to the control group. The women from both groups were at a similar age. The mean age for the breast cancer patients was 58.85 (SD 11.50) and for the healthy subject it was 60.80 (SD 7.11), *p* = 0.007. Other demographic characteristics did not differ significantly between the cases and controls. A total of 16 preselected SNPs in nine core circadian genes were genotyped and most of them did not departure from the Hardy–Weinberg equilibrium (HWE) except for three SNPs, which were not in HWE, namely: *CLOCK* rs12505266, *PER1* rs3027178, *PER3* rs2640909 in the control group (Table 2). 

### 2.1. Selected Circadian Gene Polymorphism Is Associated with Breast Cancer Risk

To determine whether the particular genotypes of core circadian genes could influence breast cancer risk, we performed genotyping analysis using TaqMan probes or High-Resolution Melting technique on DNA of 107 matched pairs tissues and 214 blood samples from breast cancer patients.

Of the 16 investigated SNPs five were found to be significantly associated with breast cancer risk. Rs2279287 is located in the 5′ UTR region of *BMAL1*, according to SNP info NIH platform (https://snpinfo.niehs.nih.gov/snpfunc.htm, Table 2). This polymorphic site is the probable point of attachment of transcription factors. The carriers of the minor allele (T) rs2279287 had a reduced predisposition to breast cancer assuming a recessive homozygous genotype OR = 0.47 (0.27–0.80) *p* = 0.02 and a dominant genetic model OR = 0.69 (0.50–0.95) *p* = 0.02. Additionally, we also found a potential protective effect (at marginal statistical significance) of minor allele (G) rs3027178 for *PER1* assuming a recessive genotype OR = 0.54 (0.28–1.08); *p* = 0.09 and a recessive genetic model OR = 0.55 (0.28–1.08); *p* = 0.08 (Table 3).

The women who had one or more protective alleles (derived at least one gene *BMAL1* rs2279287 and/or *PER1* rs3027178) had a significantly reduced breast cancer risk. OR = 0.49 (0.32–0.77) *p* = 0.002 (Table 4).

Rs10838524 is located on an intron of the *CRY2* locus. The present analysis suggested that minor allele (G) is associated at marginal statistical significance with increased breast cancer susceptibility of 65% under a recessive genotype OR = 1.65 (1.05–2.58), *p* = 0.07 and significantly of 45% under a dominant genetic model OR = 1.45 (1.00–2.10), *p* = 0.05.

Intronic rs12505266 of *CLOCK* was significantly associated with an increased predisposition to breast cancer among recessive homozygous genotypes OR = 1.22 (0.83–1.79), *p* = 0.057 and at marginal significance under a recessive genetic model OR = 1.38 (0.97–1.96), *p* = 0.07. Similar associations were observed between *PER2* missense variant rs934945 under a dominant genetic model and an increased breast cancer risk OR = 1.56 (1.09–2.23), *p* = 0.01. Potential significance of an increased predisposition to breast cancer was demonstrated for a recessive genetic model of *PER2* rs11894491 OR = 1.53 (0.93–2.50) *p* = 0.09. We found a significant association between a heterozygous genotype of *TIMELESS* rs2279665 and breast cancer risk OR = 0.69 (0.481.00) *p* = 0.02. For another SNP we found marginal significance including a recessive homozygous genotype and a recessive genetic model for *PER1* rs3027178 OR = 0.54 (0.28–1.08) *p* = 0.09; OR = 0.55 (0.28–1.08) *p* = 0.08, respectively (Table 3).

The patients having at least one or more risk alleles (among three significant SNPs *CRY2* rs10838524; *PER1* rs2735611; *PER2* rs934945) were significantly associated with an increased breast cancer risk OR = 1.66 (1.17–2.35) *p* = 0.005 (Table 4).

### 2.2. Circadian Gene Variants Are Associated with an Estrogen and Progesterone Receptor Status

In addition, to the main effects model, the control group was simultaneously compared to the breast cancer patients stratified into two groups according to their hormonal receptor status-estrogen-positive (ER+)/progesterone-positive (PR+) tumors and estrogen-negative (ER−)/progesterone-negative (PR−) tumors. The genotyping analysis were performed on DNA samples derived from 107 matched tissue pairs and 214 blood samples from breast cancer patients. Interestingly, four of 16 were significantly associated with a breast cancer risk, including two SNPs with ER+/PR+ tumors and two SNPs with both types of tumors. Recessive homozygotes of *BMAL1* rs2279287 had a significant protective effect of ER+/PR+ tumors OR = 0.45 (0.24–0.86) *p* = 0.04 in comparison to an increased risk effect of *PER1* rs2735611 OR = 3.76 (1.38–10.24) *p* = 0.01 and *PER2* rs934945 OR = 1.75 (1.16–2.63) *p* = 0.01. While recessive homozygotes of *CRY2* rs10838524 OR = 2.26 (1.03–4.94) *p* = 0.03 and *PER1* OR = 6.08 (1.80–20.56) *p* = 0.003 were significantly associated with ER-/PR- tumors Table 5.

### 2.3. A Putative Functional Effect of SNP on Circadian Gene Expression

**Figure 1:**
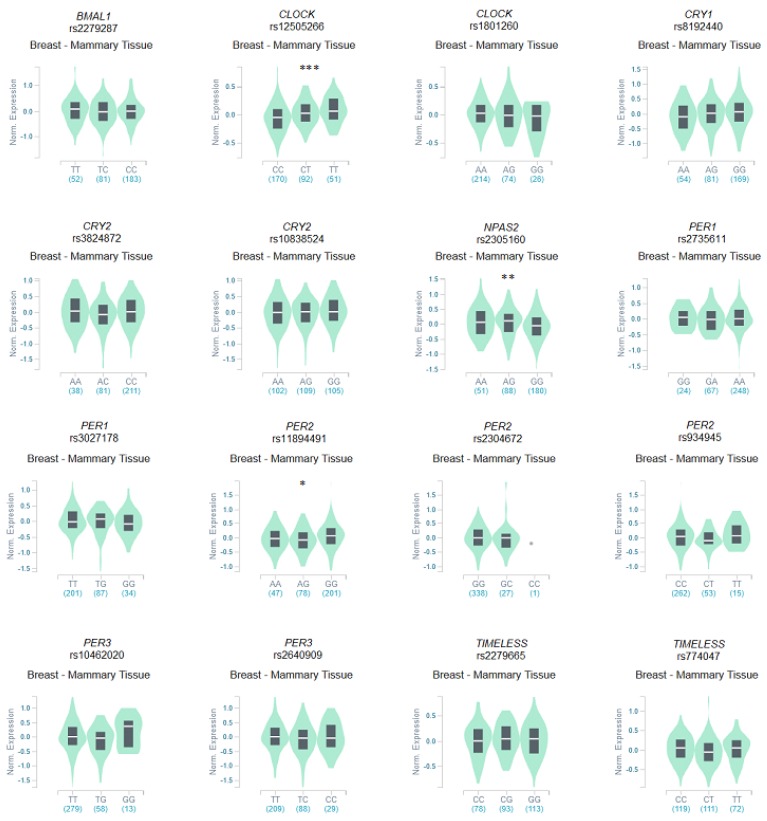
The bioinformatic analysis gene expression patterns in breast mammary tissue according to the selected genotypes of particular circadian genes using eQTL calculator on the GTex platform. * *p* < 0.05; ** *p* < 0.01; *** *p* < 0.001.

To determine whether the genotypes of SNP could alter mRNA transcription we compared data obtained from the gene expression analysis using the quantitative real-time PCR among 107 matched pairs tissues from breast cancer patients to their genotype. As shown in Table 6, we found that mRNA expression level was significantly changed according to particular gene variants, indicating that SNPs may affect transcription of these genes. The carriers who had at least one variant allele of *BMAL1* rs2279297 (*p* = 0.05) or were a recessive homozygote of *CRY2* rs3824872 (*p* = 0.0004), had a significantly decreased gene expression level in breast cancer tissue compared to the women with a dominant homozygous genotype. In contrast, the carriers of a dominant genotype of *PER3* rs10462020 had a significantly reduced level of mRNA expression in breast cancer tissue *p* = 0.02. We also observed significant differences in a transcript level in adjacent non-tumor tissues. The carriers with a variant allele had reduced gene expression of *PER1* rs2735611, (*p* = 0.04), *PER1* rs3027178 (*p* = 0.002), or an increased transcript level of *PER2* rs11894491 (*p* = 0.03). The bioinformatic analysis (GTex eQTL calculator, www.gtexportal.org) was employed to explore potential biological effects of selected SNPs of core circadian genes on their mRNA expression in normal breast-mammary tissue [8]. As shown in Figure 1, we observed that three *CLOCK* rs12505266, *p* = 0.000048; *NPAS2* rs2305160, *p* = 0.017; *PER2* rs11894491, *p* = 0.036 of the investigated genes had a significantly different gene expression pattern in breast mammary tissue according to different genotypes and two SNPs including *CLOCK* rs1801260 *p* = 0.065 and *CRY1* rs8192440 *p* = 0.069 had a different expression pattern at the marginal statistical significance (Figure 1). Only one SNP-rs11894491-showed a significant effect on a *PER2* transcript level in mammary cancer-free tissues, based on the results from our dataset and the GTex eQTL calculator. However, we identified higher gene expression in the carriers with a minor genotype in comparison to GTex platform, where the carriers with a major genotype had a higher mRNA level than the carriers with other gene variants.

## 3. Discussion

Our analysis indicates that five SNPs, *BMAL1* rs2279287, *CLOCK* rs12505266, *CRY2* rs10838524, *PER1* rs2735611, *PER2* rs934945, are significantly associated with the breast cancer risk in the Polish association study. Additionally, we observed differences in the gene expression level according to particular gene variants: *BMAL1* rs2279287, *CRY2* rs10838524, *PER1* rs2735611, rs3027178, *PER2* rs11894491, *PER3* rs10462020.

There are several studies demonstrating that alterations of circadian rhythm together with disruptions caused by shift work and exposure to light at night (LAN) may promote breast cancer development [9,10]. There are a few studies on circadian gene variants in shift workers and susceptibility to breast carcinogenesis [11,12]. Molecular epidemiological studies reveal a significant association of the polymorphic variants of circadian genes and the risk of various cancer types such as breast cancer [13,14,15,16,17], colorectal cancer [18,19,20], glioma [21], prostate cancer [22,23], hepatocellular carcinoma [24,25], lung cancer [26], non-Hodgkin’s lymphoma [27], as well as mood [28,29] and metabolic disorders [30].

Genetic variants such as SNPs play a substantial role in gene expression regulation, mRNA translation, and degradation protein structures all of which may affect gene functions and human phenotype [2]. Circadian genes play essential roles in regulation of gene expression, including cell proliferation, DNA damage repair, cell cycle control, and apoptosis. Due to their physiological role, alteration of these genes may lead to cancer development [3,4]. Considering this fact, it is sensible to assume that SNPs of circadian genes may affect cancer susceptibility, cancer cell proliferation, invasion, as well as a response to treatment and even patients’ survival [31,32,33]. Consequently, explaining the mechanism of circadian genes in cancer development will be useful in better diagnostics and improving patient’s healthcare.

In the present study, we described findings of the first approach, to the best of our knowledge, which has investigated the relationship between various polymorphic sites of circadian genes and the transcript level among breast cancer patients. The results were based on genotyping from 364 healthy women and 321 breast cancer patients. Additionally, genotyping results were analyzed according to gene expression data obtained from 107 breast cancer tissue pairs. We hypothesized that circadian gene SNPs may influence their expression level and susceptibility to breast cancer. Our results support this hypothesis. In fact, five of the 16 analyzed genetic variants were statistically significantly associated with a predisposition to breast cancer employing a dominant or recessive genetic model of inheritance. Up to now, some significant genetic variants of circadian genes have been recognized as associated with breast cancer risk [13,14,16,34,35,36,37,38].

*CLOCK* rs1801260 located on the 3′ UTR region, is one of the most common variants studied by many authors with regard to malignancies as well as other disorders [3,4]. Regarding investigations of rs1801260 and breast cancer predisposition, Hoffman et al. have indicated that under the additive genetic model, there is an increased risk of ER/PR negative breast cancer OR = 2.57 (1.14–5.82) [34]. Subsequently, CECILE study has shown that rs1801260 is linked with breast cancer risk among postmenopausal women [16]. According to SNPinfo Web Server (http://snpinfo.niehs.nih.gov/snpinfo/snpfunc.html), rs1801260 is predicted as a microRNA binding site. Further, a performed bioinformatic analysis microRNA.org—Targets and Expression (http://www.microrna.org/microrna/home.do) has revealed that rs1801260 is within the miRNA hsa-miR-141 that was identified as a probable diagnostic marker of breast cancer [39]. However, our analysis did not show a significant association between *CLOCK* and breast cancer risk. What is more, we did not demonstrate significant changes at a mRNA level under this *CLOCK* variant. We selected another rs12505266 *CLOCK* for the analysis. We found, at marginal significance, that the carriers of a minor allele T demonstrated an increased breast cancer risk in comparison to the healthy population OR = 1.21 (0.82–1.78) *p* = 0.057. At a marginal significant level, the carriers with TT genotype had a higher risk of developing ER+ tumors than ER-, OR = 1.28 (0.82–2.01). This is an intronic polymorphic site, but according to SNPinfo Web Server (http://snpinfo.niehs.nih.gov/snpinfo/snpfunc.html), rs12505266 is predicted as a transcription factor binding site of *CLOCK*, which may regulate expression of *CLOCK* gene. Furthermore, the in-silico analysis using the GTEx eQTL calculator (https://gtexportal.org/home/testyourown) [8], has shown a significantly elevated gene expression level in breast mammary tissues carrying variant-containing TT genotypes. Nevertheless, our gene expression analysis showed changes at marginal statistical significance on a mRNA level under rs12505266 *CLOCK* in adjacent non-tumor tissues, where tissues with a minor genotype had an increased transcript level of *CLOCK*. This inconsistency may be due to a smaller size population used in our study. Other epidemiological studies have indicated a negative effect of intronic rs3805151 [37], rs11932595, rs7698022, and 3′UTR SNPs rs1048004, rs1801260 [16] *CLOCK* polymorphism as well as a positive effect under the dominant genetic model of *CLOCK* rs6850524, rs13102385, rs11133391 on breast cancer susceptibility [34].

Of particular interest, the SNP rs2279287 within the *BMAL1* gene was found to be associated with a reduced breast cancer risk, among the carriers with the variant containing CT/TT genotypes than among those with the homozygous wild-type genotype (CC). A reduced risk of breast tumorigenesis was also observed in ER+ tumors compared to ER- in carriers of at least one variant allele. The polymorphic site rs2279287 as predicted on SNPinfo Web Server http://snpinfo.niehs.nih.gov/snpinfo/snpfunc.html is the probable point of transcription factor attachment, which may have an influence on *BMAL1* gene expression. Indeed, we found a significant relationship between *BMAL1* genetic polymorphism (rs2279287) and mRNA transcription. Breast cancer tissues carrying a variant containing CT/TT genotypes had a reduced level of *BMAL1* expression. In the literature, we found one publication with significant results where rs2279287 was associated with a seasonal affective disorder in Indian families [40].

Moreover, there are a few SNPs which were distinguished in Period and Cryptochrome genes. Among circadian genes, *PERs* are recognized as tumor suppressor genes, which is relevant for the process of carcinogenesis [41]. Studies on animals have shown that *PER2* knockout rodents are more prone to cancer initiation after gamma radiation in comparison to wild type mice [42,43]. Moreover, overexpression of *PER2* in MCF-7 leads to significant inhibition of breast cancer cell proliferation [41]. Therefore, most of the investigated SNPs referred to Period genes (*PER1* rs2735611, *PER1* rs3027178, *PER2* rs11894491, *PER2* rs2304672, *PER2* rs934945, *PER3* rs10462020, *PER3* rs2640909) and were incorporated into the analysis. Selected SNPs according to SNPinfo Web Server (http://snpinfo.niehs.nih.gov/snpinfo/snpfunc.html) are predicted as an exonic splicing enhancer or silencer (ESE or ESS), which may have a role in gene expression regulation. Rs934945 is located on the last exon of *PER2* and has a missense functional effect leading to the submission of Glycine-Glutamic Acid. According to NCBI Protein database, the rs934945 site is also a CRY-binding domain. Carriers who had at least one minor allele (T) had an increased breast cancer risk employing a dominant genetic model. Similar observations have been found by Dai et al., i.e., patients carrying both the *PER2* (rs934945) TT and *CLOCK* (rs3805151) CC genotypes had an increased breast cancer risk [37]. Opposite results have been demonstrated by Benna et al. showing that patients with recessive genotype *PER2* (rs934945) TT had a reduced predisposition to sarcoma and liposarcoma [32]. Additionally, other research has not indicated associations between *PER2* (rs934945) and gastric cancer [31]. Interestingly, our research partially confirmed previous findings of Benna et al. showing that *PER1* rs3027178 had a protective effect and reduced risk of soft tissue sarcoma [32]. We observed a similar effect at the statistical significance margin under the recessive genetic model. The carriers with at least one G allele may have a reduced breast cancer risk of 45%. Opposite findings have been presented by Zhang et al. where carriers with at least one minor allele (*PER1* rs3027178) among hepatocellular carcinoma patients, had a worse prognosis of recurrence-free survival [24]. A later study of Qu et al. has shown that *PER1* rs3027178 was also associated with worse overall survival of gastric cancer patients under a dominant genetic model [31]. In our study, the strongest identified association was rs2735611 in the exon 18 of *PER1*. The carriers of recessive genotype (AA) had an increased risk of breast cancer OR = 4.37 (1.78–10.69) *p* = 0.001. According to the results of gene expression, the carriers with at least one variant allele had decreased mRNA expression of *PER1*. The decreased expression level of *PER1* was observed in breast cancer tissue and associated with worse breast cancer type ER-. Among gastric cancer patients this association has not been demonstrated [31].

Together with Period genes, *CRY1* and *CRY2* form a negative arm of the circadian feedback loop and are essential for the proper functioning of circadian machinery. These genes have potential to influence expression of many biological pathways directly or indirectly [44]. For instance, knockdown of *CRY2* in MCF-7 cells increased accumulation of mutagen-induced DNA damage and altered expression of genes involved in the DNA damage response and cell cycle regulatory pathways including cyclin-dependent kinase inhibitor p21 (*CDKN1A*) and induction of cyclin D1 (*CCND1*)—an oncogene that is often overexpressed in breast cancer cases [45]. There are a few studies which focus on *CRY1* and *CRY2* polymorphism, but there are some significant gene variants associated with breast cancer risk [12,37]. In our analysis we found only one significant association among Cryptochrome genes—*CRY2* rs10838524. The patients with at least one variant allele had an increased breast cancer risk. Moreover, a minor homozygous genotype was significantly associated with breast cancer risk in the women with ER- tumors. There are no other publications where this genotype is associated with an increased cancer predisposition, but *CRY2* rs10838524 was associated with winter depression among Finnish and Swedish populations [46]. Other cryptochrome gene variants have previously been shown to be associated with a decreased breast cancer risk including GT genotype of rs1056560 in *CRY1* (OR = 0.84 95% CI = 0.71–0.99) and CC genotype of rs1401417 in *CRY2* (OR = 0.24 95% CI = 0.08–0.73) in premenopausal women [37]. However, a significant association with breast cancer risk in postmenopausal patients for three SNPs in *CRY2* has been found. GC or CC genotypes of rs1401417 (OR = 0.44 95% CI = 0.21–0.92), AG or GG genotypes of rs11038689, (OR = 0.71 95% CI = 0.51–0.99) and AA genotype of rs7123390, (OR = 0.44 95% CI = 0.22–0.86) [35]. Among Han Chinese gastric cancer patients, SNP rs1056560 in the *CRY1*, under an additive model had a protective effect on the overall survival of the patients with a HR of 0.72 (95% CI 0.58–0.88, *p* = 0.021) [31].

Another polymorphic gene with potential relevance in breast cancer development is *NPAS2*. A first case-control study on non-synonymous polymorphisms rs2305160 Ala394Thr of *NPAS2*, among 431 breast cancer women and 476 controls (most of them were Caucasians) has revealed that rs2305160 Ala/Thr genotype had a significant association with a decreased breast cancer risk in comparison to more frequent Ala/Ala genotype (OR = 0.61 95% CI = 0.46–0.81) [13]. The interesting issue is that considering the menopausal status, both groups, pre- and post-menopausal patients, had a significant relationship between heterozygous genotype and breast cancer risk, but stronger association has been observed among premenopausal women (OR = 0.44 95% CI = 0.25–0.77, *p* = 0.004) than in postmenopausal women (OR = 0.65 95% CI = 0.46–0.91) [13]. Further studies on Ala394Thr on 348 breast cancer tissue using the TaqMan allelic discrimination assay have shown a borderline, but non-significant association between homozygous Thr/Thr genotype and a poor prognosis in breast cancer survival [14]. A later study of Yuan et al. has demonstrated a significant association between rs2305160 in *NPAS2* and overall death risk in hepatocellular carcinoma patients under genetic dominant model (HR = 1.63 (1.29–2.07), *p* < 0.001) [25]. Our data analysis did not show a significant effect of this polymorphism on breast cancer among the study group. Only the bioinformatic analysis (based on GTex eQTL calculator) revealed significant differences in mRNA level according to the genotypes of rs2305160.

Among genetic association studies on circadian genes and their impact on cancer development, only one publication has demonstrated a significant relationship between polymorphic variants of *TIMELESS* (rs7302060, rs2291738) and breast cancer risk [36]. This study has reported a significant association between both tagging SNPs rs7302060, rs2291738 and breast cancer, showing that the C allele ssof rs7302060 has been linked to reduction of breast cancer risk (OR = 0.54 95% CI = 0.54–0.99). A similar observation was made for two genotypes CC of rs730260 (OR = 0.35 95% CI = 0.16–0.78) and GG of rs2291738 (OR = 0.45 95% CI = 0.217–0.97). Both had an association with a decreased breast cancer risk among ER/PR positive tumors [36]. Our analysis revealed that a heterozygous genotype of rs2279665 is linked to a reduced predisposition to breast cancer risk also in the case of ER+/PR+ tumors.

Moreover, the recent GWAS study conducted by Mocellin et al. has shown a highly significant association between the breast cancer risk and circadian pathway variation (*p*-value 1.9 × 10^−6^) The top genes and number of SNPs involved in cancer development were *RORA* and *RORB* (eight SNPs and five SNPs, respectively); other significant genes with single SNPs were *PER1* (two SNPs), *ARNTL*, *CRY2*, *CLOCK*, *CRY1*, and *RORC*, in order of significance. A similar observation has been also found for ER- breast tumors [27].

A potential limitation of our study was the relatively small size of the study group. Additionally, we were not able to perform prognostic analysis stratified by circadian SNPs, according to patients’ overall survival because of the lack of relevant data. Therefore, associations between circadian gene polymorphisms and BC should be treated with caution and validated on a larger population.

## 4. Methods and Materials

### 4.1. Study Population and Biological Materials

A total of 321 Polish women, all of Caucasian origin, with a newly and histologically diagnosed BC and without previous chemotherapy were included in this association study. The patients were derived from two medical centers in Poland: Clinic of Oncologic Surgery at the Medical University of Gdansk and the Copernicus Memorial Hospital, Lodz, Poland. The breast cancer patients were hospitalized over the years 2006–2015. The study group consisted of breast cancer patients who were consecutively admitted to the Clinics and agreed to donate the biological material for scientific purposes. Biological material consisted of 107 BC tissue pairs tumor tissue and tumor-adjacent (cancer-free) tissue (0.6 × 0.6 cm from the same patient) and 214 blood samples (7.5 mL). Matched BC tissue samples were derived from a group of patients hospitalized at Clinic of Oncologic Surgery at the Medical University of Gdansk while blood samples were derived from a group of patients hospitalized at the Copernicus Memorial Hospital. All the tissue specimens were collected during the tumor removal surgery and evaluated by pathologists to confirm the diagnosis of both tumor and adjacent tissue (the second one was collected close to the resection margin). Then the samples of BC tissue pairs were frozen at −70 °C and deposited in Bank of Frozen Tissues and Genetic Specimens at Medical University of Gdansk for a further molecular analysis. The blood samples (7.5 mL) were collected from the patients into heparinized test tubes and separated by centrifugation into a buffy coat for DNA isolation. Each fraction was stored at −20 °C until a molecular analysis at NIOM.

The control group consisted of 364 of healthy women who were gathered for projects carried out at NIOM between 2008 and 2014. Eligible controls at above 50 years of age and with lack of shift work history were selected from general population, who voluntarily agreed to donate biological material for scientific purposes. Blood samples (7.5 mL) were collected from each healthy volunteer into heparinized test tubes and separated by centrifugation into the buffy coat for DNA isolation. Each fraction was stored at −20 °C until a molecular analysis at NIOM. The women from the control group who were reported to work in shifts (at the time of recruitment) were not included in the study, because shiftwork that involves circadian disruption has been classified by the International Agency for Research on Cancer as a probable human carcinogen group 2A [47,48].

Basic epidemiological characteristics (age, BMI, smoking, and menopausal status) were collected using individual questionnaires (from the BC patients and the control group), whereas clinical data (receptor status, tumor stage and grade, histological type of tumor) were obtained from medical records of both groups of the BC patients (Table 1).

### 4.2. Ethics Declaration

This study was conducted in compliance with the Declaration of Helsinki and was approved by the relevant Local Ethics Committees (Independent Ethics Committee at Medical University of Gdansk, resolution No. NKEBN/781/2005, on 15 December 2005 and Ethical Institutional Review Board at the Nofer Institute of Occupational Medicine, resolution No. 4/2019; on 4 April 2019. We obtained a written consent of the participants to deposit their biological material in the Bank of Frozen Tissues and Genetic Specimens of the Medical University of Gdansk (tissue specimens) and in the NIOM (blood specimens) to use them in further analyses.

### 4.3. DNA Isolation Procedures

Total genomic DNA from the tissue pairs (adjacent non-tumor breast tissue and breast cancer tissue) and buffy coat were isolated by the DNeasy Blood and Tissue KIT (Qiagen, Hilden, Germany) according to the manufacturer’s protocol in order to evaluate SNPs in circadian genes. The amount of the total extracted DNA was determined in microplates using the spectrophotometric method (MultiscanTM Go Microplate Spectrophotometer, Thermo Fisher; Waltham, MA, USA).

### 4.4. SNP Selection, Genotyping, and Gene Expression.

Potential functional SNPs that were included had to meet the following criteria: SNPs of core circadian genes were selected based on their position within the gene, predicted function according to SNPinfo Web Server (https://snpinfo.niehs.nih.gov/snpfunc.htm); validated SNPs with minor allele >10% in the Caucasian population according to the SNP base in the National Center for Biotechnology Information, https://www.ncbi.nlm.nih.gov, and on the basis of relevant literature [3,4]. Finally, a total of 16 functional SNPs in nine core circadian genes were selected including non-synonymous variation, potential transcription factor binding site, potential splice site, or potential miRNA- binding site (Table 2).

Allelic discrimination for *CRY2* (rs10838524), *PER3* (rs10462020), *PER1* (rs2735611), *PER2* (rs934945), *PER2* (rs2304672), *NPAS2* (rs2305160), *TIMELESS* (rs2279665) was conducted using the TaqMan SNP Genotyping Assays (Thermo Fisher Scientific, Waltham, MA, USA) and for genes *CLOCK* (rs12505266), *CRY2* (rs3824872) *PER1* (rs3027178) *PER2* (rs11894491) PER3 (rs2640909) TIMELESS (rs774047) we used the TaqMan probes and primers (Eurofins Genomics, Ebersberg, Germany). Genotyping was performed using the Real-Time PCR method and the Light Cycler 96 Roche (Roche, Basel Switzerland) and FastStart DNA probes master mix (Roche, Basel Switzerland). PCR reactions were carried out with 10 ng of DNA in a final volume of 10 μL. For genes *CLOCK* (rs1801260), *BMAL1* (rs2279287), *CRY1* (rs8192440) we used the High-Resolution Melt Curve technique and restriction fragment length polymorphism-polymerase chain reaction (RFLP-PCR) for genotype verification. Primers were designed with Beacon Designer 7.01 (PREMIER Biosoft International, Palo alto, CA, USA) according to the GenBank^®^ genetic sequence database. HRM assay was performed on Light Cycler 96 Roche (Roche, Basel, Switzerland) using 10 ng of DNA in a final volume of 20 μL and SsoFastTM EvaGreen Supermix (Bio-Rad Laboratories, Inc, Hercules, CA, USA). Genotyping was performed using the control DNA samples with known genotypes and a negative control.

### 4.5. Gene Expression

Gene expression data obtained from the 107 tissue pairs of breast cancer patients were compared with the results of allelic discrimination in order to check the influence of selected SNPs on the transcriptome of particular circadian genes. Gene expression assay has been described in detail in our previous work [47] Our results from allelic discrimination were also compared with The Genotype-Tissue Expression (GTEx) using the eQTL calculator (https://gtexportal.org/home/testyourown) [8]. Results of the in silico analysis were obtained using the following data source: GTEx Analysis Release V8 (dbGaP Accession phs000424.v8.p2) on 14 October, 2019. The clinical material used in GTEx platform consisted of 396 Breast Mammary Tissues from the women between 20 and 79 years of age and of mixed ethnicity. GTEx platform does not provide eQTL analysis for cancer tissues, therefore we could not perform in silico analysis for BC tissues.

### 4.6. Statistics Analysis

Means with standard deviation and frequencies of the basic features were calculated. Normality of the data was evaluated with the Shapiro–Wilk test. The Chi-square test was performed to determine any discrepancies of distribution from the Hardy–Weinberg Equilibrium (HWE). Odds ratios (ORs) and their corresponding 95% confidence intervals (CI) were calculated by the logistic regression analysis in order to measure the strength of association of selected circadian genes polymorphisms and their interactions with breast cancer risk. The *p*-value was adjusted by confounding variables (age, BMI, smoking status, menopausal status). Additionally, two genetic models: dominant genetic model (genotypes with a presence of at least one copy of the minor allele vs. dominant homozygous genotype) and recessive genetic model (recessive homozygous genotype vs. genotypes with a presence of at least one copy of the major allele) were considered. Mean Normalized Expression (MNE) was calculated previously [49]. The obtained data from gene expression were analyzed according to the best fitting genetic models using the Student’s *t*-test. All the performed analyses were calculated using STATISTICA 12 software package (Statsoft, Tulsa, OK, USA).

## 5. Conclusions

Our association study indicated that circadian gene variants, including *BMAL1* rs2279287, *CLOCK* rs12505266, *CRY2* rs10838524, *PER1* rs2735611, *PER2* rs934945, rs11894491, may be associated with breast carcinogenesis. This relationship seems to have a reflection in a biological effect because polymorphisms may influence gene expression, protein function like protein-protein interactions, or mRNA stability. This is consistent with the results obtained in our previous studies [49,50]. However, due to the limited size of the group, the association between functional SNPs in circadian genes and breast cancer susceptibility needs to be interpreted with caution and validated in a larger population. Finally, the majority of breast cancer association studies and circadian genes SNPs were conducted among a Caucasian population. Importantly, it should be remembered, that variability of alleles exists in populations, so allele variants of specific genes are frequently observed in specific populations like Caucasian or Asian populations.

## Figures and Tables

**Table 1:** Selected demographic and clinical characteristics of the breast cancer patients and healthy population in the association study.

Characteristics	Breast Cancer Patients (*n* = 321)	Healthy Population (*n* = 364)	*p*-Value
Age (years), mean (SD)	58.85 (11.50)	60.80 (7.11)	**0.007 ^1^**
BMI (kg/m^2^); mean (SD)	27.13 (4.67)	27.08 (4.62)	0.90 ^1^
Menopausal status			
Pre-menopause	77	37	**0.00001 ^2^**
Post-menopause	233	325	
Unknown	11	2	
Smoking status			
Never smokers	249	225	**0.00001 ^2^**
Past/Current smokers	69	138	
Unknown	3	1	
Tumor stage			
I	157		
II	83		
III	58		
IV	9		
Unknown	14		
Nodal status			
Yes	123		
No	183		
Unknown	15		
Histological type			
*Carcinoma ductale*	174		
*Carcinoma lobulare*	30		
Mix type of carcinoma	115		
Unknown	2		
Differentiation Grade (G)			
Well	27		
Moderate	151		
Poor	104		
Unknown	39		
Estrogen receptor status (ER)			
Positive	227		
Negative	80		
Unknown	14		
Progesterone Receptor status (PR)			
Positive	192		
Negative	115		
Unknown	14		
HER2 receptor status			
positive	139		
negative	161		
Unknown	21		

Notes: significant *p*-values are marked in bold. ^1^ The *p* values were calculated using the Student’s *t*-test. ^2^ The *p* values were calculated using the Pearson Chi-Square test.

**Table 2:** Selected circadian genes’ single nucleotide polymorphisms subject to analysis and their predicted functions.

Gene	SNP ID	Chromosome Position	Allele Major/Minor	Region	MAF Global	MAF Control Group	Probable Function	Genotyping
*BMAL1*	rs2279287	chr11:13298485	C/T	5′UTR	0.43	0.37	TFBS	HRM
*CLOCK*	rs12505266	chr4: 56412169	C/T	intron	0.33	0.43 ^1^	TFBS	TaqMan probes
*CLOCK*	rs1801260	chr4:56301369	A/G	3′UTR	0.25	0.33	probable miRNA binding site hsa-miR-141	HRM
*CRY1*	rs8192440	chr12:107395106	C/T	exon 5	0.21	0.36	Splicing (ESE or ESS) Gly/Gly	HRM
*CRY2*	rs3824872	chr11:45905605	C/A	near gene	0.46	0.24	TFBS	TaqMan probes
*CRY2*	rs10838524	chr11:45870177	A/G	intron	0.33	0.47	N/A	TaqMan probes
*NPAS2*	rs2305160	chr2:101591304	G/A	intron	0.2	0.36	Splicing (ESE or ESS) Thr/Ala	TaqMan probes
*PER1*	rs2735611	chr17:8048283	G/A	exon 18	0.42	0.13	Splicing (ESE or ESS) Gly/Gly	TaqMan probes
*PER1*	rs3027178	chr17: 8053085	T/G	exon 5	0.29	0.25 ^1^	Splicing (ESE or ESS) Thr/Thr	TaqMan probes
*PER2*	rs11894491	chr2:239198325	G/A	intron	0.24	0.34	TFBS	TaqMan probes
*PER2*	rs2304672	chr2 239186589	G/C	5′UTR	0.09	0.13	Splicing (ESE or ESS)	TaqMan probes
*PER2*	rs934945	chr2:239155053	C/T	exon 23	0.22	0.13	CRY binding domain Gly/Glu	TaqMan probes
*PER3*	rs10462020	chr1:7880683	T/G	exon 15	0.12	0.19	Splicing (ESE or ESS) Val/Gly	TaqMan probes
*PER3*	rs2640909	chr1:7830057	T/C	exon 18	0.18	0.30 ^1^	Met/Thr	TaqMan probes
*TIMELESS*	rs2279665	chr12:56827694	G/C	exon 3	0.46	0.43	Splicing (ESE or ESS) Leu/Leu	TaqMan probes
*TIMELESS*	rs774047	chr12:56815922	C/T	exon 20	0.49	0.43	Splicing (ESE or ESS) Gln/Arg	TaqMan probes

^1^ Not in HWE; HRM High Resolution Melt; TFBS Transcription factor binding site; ESE Exonic splicing enhancer; ESS Exonic splicing silencer; N/A not applicable.

**Table 3:** Association between the selected single nucleotide polymorphisms (SNPs) of circadian genes with breast cancer risk.

Gene	Genotype	Ctrl	Cases	OR (95%CI)	*p*-Value ^3^
*BMAL1* rs2279287	CC	152	159	Ref.	
	CT	155	127	0.76 (0.54–1.07)	0.56
	TT	57	28	0.47 (0.27–0.80)	**0.02**
	CT + TT ^1^	212	155	0.69 (0.50–0.95)	**0.02**
	TT ^2^	57	28	0.53 (0.32–0.89)	**0.02**
	unknown	0	7		
*CLOCK* rs1801260	AA	160	146	Ref.	
	AG	168	125	0.79 (0.56–1.12)	0.18
	GG	34	40	1.03 (0.60–1.78)	0.57
	AG + GG ^1^	202	165	0.84 (0.60–1.16)	0.28
	GG ^2^	34	40	1.16 (0.69–1.94)	0.58
	unknown	2	10		
*CLOCK* rs12505266	CC	145	134	Ref.	
	CT	125	88	0.75 (0.51–1.10)	**0.03**
	TT	94	98	1.22 (0.83–1.79)	0.057
	TT + CT ^1^	219	186	0.95 (0.69–1.31)	0.75
	TT ^2^	94	98	1.38 (0.97–1.96)	0.07
	unknown	0	1		
*CRY1* rs8192440	CC	148	105	Ref.	
	CT	165	118	0.98 (0.68–1.41)	0.94
	TT	49	34	0.98 (0.58–1.66)	0.97
	CT + TT ^1^	214	152	0.98 (0.69–1.38)	0.89
	TT ^2^	49	34	0.99 (0.60–1.62)	0.97
	unknown	2	64		
*CRY2* rs10838524	AA	104	69	Ref.	
	AG	177	157	1.36 (0.91–2.01)	0.73
	GG	83	91	1.65 (1.05–2.58)	0.07
	AG + GG ^1^	260	248	1.45 (1.00–2.10)	**0.05**
	GG ^2^	83	91	1.35 (0.93–1.95)	0.11
	unknown	0	4		
*CRY2* rs3824872	CC	210	195	Ref.	
	CA	133	108	0.95 (0.68–1.34)	0.73
	AA	21	17	1.06 (0.53–2.13)	0.82
	CA + AA ^1^	154	125	0.97 (0.70–1.34)	0.84
	AA ^2^	21	17	1.08 (0.54–2.14)	0.83
	unknown	0	1		
*PER1* rs2735611	GG	278	222	Ref.	
	GA	79	69	0.97 (0.66–1.44)	**0.01**
	AA	7	22	4.34 (1.77–10.67)	**0.001**
	GA + AA ^1^	86	91	1.23 (0.85–1.77)	0.26
	AA ^2^	7	22	4.37 (1.78–10.69)	**0.001**
	unknown	0	8		
*PER1* rs3027178	TT	214	180	Ref.	
	TG	120	120	0.96 (0.68–1.35)	0.24
	GG	30	18	0.54 (0.28–1.08)	0.09
	TG + GG ^1^	150	138	0.88 (0.63–1.22)	0.43
	GG ^2^	30	18	0.55 (0.28–1.08)	0.08
	unknown	0	3		
*PER2* rs934945	CC	275	215	Ref.	
	CT	82	91	1.52 (1.05–2.19)	0.87
	TT	7	11	2.08 (0.74–5.83)	0.32
	CT + TT ^1^	89	102	1.56 (1.09–2.23)	**0.01**
	TT ^2^	7	11	1.85 (0.66–5.17)	0.24
	unknown	0	4		
*PER2* rs2304672	GC	280	254	Ref.	
	GC	77	60	0.86 (0.57–1.29)	0.31
	CC	7	3	0.3 (0.06–1.46)	0.16
	GC + CC^1^	84	63	0.81 (0.54–1.20)	0.28
	CC ^2^	7	3	0.31 (0.06–1.50)	0.14
	unknown	0	4		
*PER2* rs11894491	GG	156	139	Ref.	
	GA	170	136	0.94 (0.67–1.32)	0.15
	AA	38	45	1.48 (0.88–2.50)	0.09
	AA + GA ^1^			1.03 (0.75–1.42)	0.86
	AA ^2^	38	45	1.53 (0.93–2.50)	0.09
	unknown	0	1		
*PER3* rs10462020	TT	241	206	Ref.	
	TG	110	95	1.01 (0.71–1.43)	0.19
	GG	13	19	1.98 (0.88–4.44)	0.10
	TG + GG ^1^	123	114	1.09 (0.78–1.53)	0.60
	GG ^2^	13	19	1.97 (0.89–4.39)	0.10
	unknown	0	1		
*PER3* rs2640909	TT	193	162	Ref.	
	TC	125	106	0.98 (0.69–1.40)	0.33
	CC	45	52	1.38 (0.85–2.23)	0.16
	TC + CC ^1^	170	158	1.08 (0.79–1.49)	0.63
	CC ^2^	45	52	1.39 (0.88–2.20)	0.16
	unknown	1	1		
*NPAS2* rs2305160	GG	150	131	Ref.	
	GA	166	136	0.92 (0.65–1.31)	0.38
	AA	48	49	1.16 (0.71–1.89)	0.41
	GA + AA ^1^	214	185	0.98 (0.71–1.35)	0.89
	AA ^2^	48	49	1.21 (0.77–1.90)	0.41
	unknown	0	5		
*TIMELESS* rs774047	CC	118	99	Ref.	
	CT	183	155	0.98 (0.68–1.41)	0.35
	TT	63	66	1.32 (0.83–2.10)	0.18
	CT + TT ^1^	246	221	1.06 (0.76–1.50)	0.72
	TT ^2^	63	66	1.33 (0.88–2.01)	0.17
	unknown	0	1		
*TIMELESS* rs2279665	GG	114	112	Ref.	
	GC	188	133	0.69 (0.48–1.00)	**0.02**
	CC	62	62	1.04 (0.66–1.66)	0.29
	GC + CC ^1^	250	195	0.78 (0.55–1.10)	0.15
	CC ^2^	62	62	1.29 (0.86–1.95)	0.22
	unknown	0	14		

The significant values are marked in bold (*p* ≤ 0.05). OR, odds ratio; CI, confidence interval. ^1^ A dominant genetic model. ^2^ A recessive genetic model. ^3^ *p*-value adjusted by age, BMI, smoking status, menopausal status.

**Table 4:** Association between risk or protective SNPs of circadian genes with breast cancer predisposition.

Gene	Ctrl	Cases	OR (95%CI)	p-Value ^3^
One risk allele ^1^	90	100	1.53 (1.07–2.20)	**0.020**
Two risk alleles ^1^	2	12	8.21 (1.71–39.42)	**0.009**
One or two risk alleles ^1^	92	112	1.67 (1.17–2.37)	**0.004**
One or more risk ^1^ alleles	93	112	1.66 (1.17–2.35)	**0.005**
One protective allele ^2^	77	41	0.48 (0.30–0.77)	**0.002**
Two protective alleles ^2^	5	3	0.71 (0.16–3.08)	0.650
One or two protective alleles ^2^	82	44	0.49 (0.32–0.77)	**0.002**

The significant values are marked in bold (*p* ≤ 0.05). OR, odds ratio; CI, confidence interval. ^1^ risk allele: *CRY2* rs10838524; *PER1* rs2735611; *PER2* rs934945. ^2^ protective alleles: *BMAL1* rs2279287; *PER1* rs3027178. ^3^ *p*-value adjusted by age, BMI, smoking status, menopausal status.

**Table 5:** Selected SNPs of circadian genes and breast cancer risk according to the hormonal receptor status.

			ER+/PR+			ER-/PR-		
Gene	Genotypes	Ctrl	Cases	OR (95%CI)	*p*-Value ^3^	Cases	OR (95%CI)	*p*-Value ^3^
*BMAL1* rs2279287	CC	152	94	Ref.		37	Ref.	
	CT	155	76	0.74 (0.50–1.10)	0.67	30	0.83 (0.46–1.50)	0.46
	TT	57	15	0.45 (0.24–0.86)	0.04	6	0.42 (0.15–1.19)	0.13
	CT + TT ^1^	212	91	0.66 (0.46–0.97)	0.03	36	0.73 (0.42–1.28)	0.27
	TT ^2^	57	15	0.52 (0.28–0.97)	0.04	6	0.46 (0.17–1.25)	0.13
*CLOCK* rs1801260	AA	160	88	Ref.		33	Ref.	
	AG	168	76	0.76 (0.51–1.13)	0.52	26	0.68 (0.37–1.26)	0.07
	GG	34	18	0.77 (0.40–1.5)	0.70	14	1.46 (0.64–3.35)	0.16
	AG + GG ^1^	202	94	0.76 (0.52–1.11)	0.16	40	0.83 (0.47–1.44)	0.50
	GG ^2^	34	18	0.88 (0.47–1.66)	0.70	14	1.74 (0.80–3.82)	0.17
*CLOCK* rs12505266	CC	145	77	Ref.		32	Ref.	
	CT	125	52	0.81 (0.52–1.27)	0.11	24	0.80 (0.41–1.56)	0.48
	TT	94	59	1.28 (0.82–2.01)	0.09	20	1.00 (0.51–1.95)	0.73
	TT + CT ^1^	219	111	1.01 (0.69–1.48)	0.95	44	0.89 (0.51–1.55)	0.68
	TT ^2^	94	59	1.40 (0.93–2.11)	0.10	20	1.09 (0.59–2.02)	0.78
*CRY1* rs8192440	CC	148	69	Ref.		22	Ref.	
	CT	165	61	0.83 (0.54–1.28)	0.24	30	1.13 (0.59–2.17)	0.51
	TT	49	24	1.16 (0.64–2.09)	0.39	6	0.82 (0.30–2.26)	0.59
	CT + TT ^1^	214	85	0.91 (0.61–1.36)	0.63	36	1.06 (0.57–1.97)	0.86
	TT ^2^	49	24	1.27 (0.73–2.20)	0.39	6	0.76 (0.29–1.98)	0.58
*CRY2* rs10838524	AA	104	38	Ref.		14	Ref.	
	AG	177	96	1.52 (0.96–2.43)	0.39	37	1.32 (0.64–2.73)	0.66
	GG	83	51	1.67 (0.98–2.84)	0.18	24	2.26 (1.03–4.94)	**0.03**
	AG + GG ^1^	260	147	1.57 (1.01–2.44)	0.05	61	1.60 (0.81–3.16)	0.17
	GG ^2^	83	51	1.26 (0.82–1.93)	0.29	24	1.87 (1.02–3.42)	**0.04**
*CRY2* rs3824872	CC	210	112	Ref.		50	Ref.	
	CA	133	68	1.07 (0.72–1.58)	0.68	24	0.66 (0.36–1.21)	0.90
	AA	21	8	0.91 (0.38–2.18)	0.78	2	0.50 (0.10–2.36)	0.53
	CA + AA ^1^	154	76	1.05 (0.72–1.53)	0.81	26	0.64 (0.36–1.15)	0.14
	AA ^2^	21	8	0.89 (0.38–2.09)	0.79	2	0.57 (0.12–2.69)	0.48
*PER1* rs2735611	GG	278	131	Ref.		52	Ref.	
	GA	79	40	0.93 (0.58–1.47)	0.03	16	0.89 (0.45–1.77)	**0.02**
	AA	7	12	3.76 (1.38–10.24)	0.01	6	6.08 (1.80–20.56)	**0.003**
	GA+AA ^1^	86	52	1.14 (0.75–1.75)	0.54	22	1.24 (0.67–2.28)	0.50
	AA ^2^	7	12	3.82 (1.41–10.35)	0.01	6	6.23 (1.86–20.9)	**0.003**
*PER1* rs3027178	TT	214	113	Ref.		45	Ref.	
	TG	120	62	0.87 (0.58–1.30)	0.79	27	0.75 (0.41–1.38)	0.57
	GG	30	13	0.65 (0.31–1.40)	0.35	4	0.35 (0.10–1.31)	0.17
	TG + GG ^1^	150	75	0.82 (0.56–1.21)	0.32	31	0.67 (0.37–1.19)	0.17
	GG ^2^	30	13	0.69 (0.33–1.45)	0.33	4	0.40 (0.11–1.43)	0.16
*PER2* rs934945	CC	275	122	Ref.		52	Ref.	
	CT	82	57	1.73 (1.13–2.63)	0.59	19	1.44 (0.76–2.71)	0.64
	TT	7	6	2.02 (0.6–6.76)	0.48	7	3.16 (0.74–13.42)	0.19
	CT + TT ^1^	89	63	1.75 (1.16–2.63)	0.01	202	1.57 (0.86–2.87)	0.14
	TT ^2^	7	6	1.71 (0.52–5.68)	0.38	7	2.88 (0.69–12.08)	0.15
*PER2* rs2304672	GC	280	154	Ref.		57	Ref.	
	GC	77	30	0.71 (0.43–1.16)	0.52	17	1.01 (0.52–1.95)	0.70
	CC	7	1	0.24 (0.03–1.98)	0.24	1	0.63 (0.07–5.37)	0.67
	GC + CC ^1^	84	31	0.66 (0.41–1.07)	0.10	18	0.97 (0.51–1.85)	0.92
	CC ^2^	7	1	0.25 (0.03–2.11)	0.20	1	0.63 (0.07–5.33)	0.67
*PER2* rs11894491	GG	156	82	Ref.		30	Ref.	
	GA	170	79	0.94 (0.63–1.4)	0.15	37	1.11 (0.62–1.98)	0.95
	AA	38	27	1.61 (0.88–2.94)	0.08	9	1.28 (0.49–3.30)	0.66
	AA + GA ^1^	208	106	1.05 (0.72–1.53)	0.80	46	1.14 (0.65–1.98)	0.65
	AA ^2^	38	27	1.66 (0.94–2.93)	0.08	9	1.21 (0.49–2.97)	0.68
*PER3* rs10462020	TT	241	121	Ref.		48	Ref.	
	TG	110	58	0.99 (0.66–1.49)	0.37	23	1.15 (0.63–2.08)	0.54
	GG	13	8	1.69 (0.63–4.50)	0.29	5	2.2 (0.58–8.44)	0.29
	TG + GG ^1^	123	66	1.05 (0.71–1.55)	0.82	28	1.23 (0.70–2.18)	0.47
	GG ^2^	13	8	1.69 (0.64–4.47)	0.29	5	2.10 (0.56–7.9)	0.27
*PER3* rs2640909	TT	193	97	Ref.		40	Ref.	
	TC	125	61	0.93 (0.62–1.41)	0.51	23	0.79 (0.42–1.5)	0.11
	CC	45	30	1.16 (0.66–2.03)	0.50	13	1.79 (0.83–3.86)	0.06
	TC + CC ^1^	170	91	0.99 (0.69–1.44)	0.98	36	1.03 (0.59–1.79)	0.92
	CC ^2^	45	30	1.19 (0.7–2.04)	0.52	13	1.95 (0.94–4.07)	0.07
*NPAS2* rs2305160	GG	150	76	Ref.		30	Ref.	
	GA	166	81	0.97 (0.65–1.46)	0.60	33	1.05 (0.57–1.92)	0.93
	AA	48	28	1.17 (0.67–2.05)	0.52	11	1.04 (0.44–2.46)	0.96
	GA + AA ^1^	214	109	1.02 (0.7–1.49)	0.93	44	1.05 (0.59–1.85)	0.87
	AA ^2^	48	28	1.19 (0.70–2.00)	0.52	11	1.02 (0.46–2.26)	0.96
*TIMELESS* rs774047	CC	118	59	Ref.		22	Ref.	
	CT	183	94	1.02 (0.67–1.55)	0.81	38	1.05 (0.56–1.99)	0.65
	TT	63	35	1.13 (0.66–1.96)	0.64	16	1.44 (0.65–3.17)	0.34
	CT + TT ^1^	246	129	1.05 (0.70–1.56)	0.83	54	1.15 (0.63–2.10)	0.65
	TT ^2^	63	35	1.12 (0.69–1.83)	0.64	16	1.39 (0.70–2.76)	0.34
*TIMELESS* rs2279665	GG	114	71	Ref.		20	Ref.	
	GC	188	75	0.64 (0.42–0.98)	0.08	38	1.13 (0.58–2.20)	0.51
	CC	62	32	0.82 (0.48–1.42)	0.91	16	1.86 (0.83–4.17)	0.11
	GC + CC ^1^	250	107	0.69 (0.46–1.02)	0.06	54	1.30 (0.69–2.43)	0.42
	CC ^2^	62	32	1.06 (0.65–1.74)	0.81	16	1.72 (0.87–3.40)	0.12

The significant values are marked in bold (*p* ≤ 0.05). OR, odds ratio; CI, confidence interval. ^1^ A dominant genetic model. ^2^ A recessive genetic model. ^3^ *p*-value adjusted by age, BMI, smoking status, menopausal status.

**Table 6:** Gene expression of the selected circadian genes according to gene variants in the breast cancer tissues and adjacent non-tumor tissues.

Gene	Adjacent Non-Tumor Tissues					Breast Cancer Tissues				
	Mean ± SD	N ^1^	Mean ± SD	N ^1^	*p*-Value	Mean ± SD	N^1^	Mean ± SD	N^1^	*p*-Value ^2^
*BMAL1* rs2279287	CC		CT + TT			CC		CT + TT		
	1.78 ± 0.37	41	1.8 ± 0.29	38	0.82	1.88 ± 0.34	50	1.75 ± 0.30	45	**0.05**
*CLOCK* rs1801260	AA + AG		GG			AA + AG		GG		
	1.82 ± 0.34	72	1.97 ± 0.24	11	0.17	2.03 ± 0.41	82	2.00 ± 0.40	15	0.79
*CLOCK* rs12505266	CC + CT		TT			CC + CT		TT		
	1.79 ± 0.37	57	1.91 ± 0.27	34	0.09	2.03 ± 0.42	67	2.01 ± 0.4	38	0.80
*CRY1* rs8192440	CC + CT		TT			CC + CT		TT		
	2.1 ± 0.38	70	2.05 ± 0.25	8	0.74	2.13 ± 0.38	75	1.91 ± 0.56	10	0.10
*CRY2* rs10838524	CC + CA		AA			CC + CA		AA		
	2.37 ± 0.39	91	2.16 ± 0.66	5	0.26	1.98 ± 0.45	99	1.27 ± 0.46	6	**0.0004**
*CRY2* rs3824872	AG + GG		AA			AG + GG		AA		
	2.38 ± 0.42	75	2.27 ± 0.38	17	0.34	1.97 ± 0.48	83	1.81 ± 0.42	19	0.18
*PER1* rs3027178	TT		TG + GG			TT		TG + GG		
	2.12 ± 0.45	58	1.77 ± 0.37	40	0.002	1.63 ± 0.56	64	1.66 ± 0.94	41	0.88
*PER1* rs2735611	GA + GG		AA			GA + GG		AA		
	2.14 ± 0.45	83	1.81 ± 0.47	9	0.04	1.67 ± 0.56	88	1.41 ± 0.67	12	0.14
*PER2* rs934945	CT + TT		CC			CT + TT		CC		
	2.2 ± 0.45	33	2.12 ± 0.31	60	0.30	2.04 ± 0.4	38	1.97 ± 0.46	63	0.45
*PER2* rs11894491	GA + GG		AA			GA + GG		AA		
	2.11 ± 0.36	86	2.42 ± 0.23	11	0.007	1.99 ± 0.44	92	2.06 ± 0.49	12	0.61
*PER2* rs2304672	GG		GC + CC			CC		CG + GG		
	2.16 ± 0.36	75	2.1 ± 0.39	18	0.61	1.99 ± 0.45	82	2.05 ± 0.4	19	0.57
*PER3* rs10462020	TT		TG + GG			TT		TG + GG		
	2.42 ± 0.34	70	2.5 ± 0.38	26	0.33	2.14 ± 0.52	76	2.4 ± 0.55	28	**0.02**
*PER3* rs2640909	TC + TT		CC			TC + TT		CC		
	2.44 ± 0.36	65	2.46 ± 0.35	31	0.78	2.22 ± 0.51	71	2.18 ± 0.61	33	0.76
*NPAS2* rs2305160	GG + GA		AA			GG + GA		AA		
	1.29 ± 0.54	66	1.56 ± 0.66	15	0.06	1.44 ± 0.64	70	1.55 ± 0.65	17	0.53
*TIMELESS* rs2279665	GG		GC + CC			GG		GC + CC		
	1.34 ± 0.48	26	1.33 ± 0.57	59	0.97	1.66 ± 0.64	30	1.66 ± 0.54	72	0.98
*TIMELESS* rs774047	CC + CT		TT			CC + CT		TT		
	1.31 ± 0.53	73	1.44 ± 0.59	15	0.38	1.69 ± 0.57	80	1.58 ± 0.58	25	0.39

The significant values are marked in bold (p ≤ 0.05); ^1^ N reflects the number of tissues with complete gene expression and genotyping results, ^2^ *p*-value obtained by means of the Student’s *t*-test.

## Abbreviations

BC	Breast cancer
SNP	Single Nucleotide Polymorphism
ER	Estrogen Receptor
PR	Progesterone Receptor
HER2	Human Epidermal Growth Factor Receptor 2
GWAS	Genome-Wide Association Study
OR	Odds Ratio
SD	Standard Deviation
CI	Confidence Intervals
HWE	Hardy–Weinberg Equilibrium
MNE	Mean Normalized Expression
GTex	The Genotype-Tissue Expression
eQTL	Expression quantitative trait loci
HRM	High Resolution Melt
TFBS	Transcription factor binding site
ESS	exonic splicing silencer
ESE	exonic splicing enhancer
LAN	light at night
